# Edentulism and quality of life among older Ghanaian adults

**DOI:** 10.1186/s12903-015-0034-6

**Published:** 2015-04-09

**Authors:** Sandra A Hewlett, Alfred E Yawson, Benedict NL Calys–Tagoe, Nirmala Naidoo, Pamela Martey, Somnath Chatterji, Paul Kowal, George Mensah, Nadia Minicuci, Richard B Biritwum

**Affiliations:** 1 Department of Restorative Dentistry, University of Ghana Dental School, College of Health Sciences, P. O. Box KB 460, Korle-Bu, Accra, Ghana; 2 Department of Community Health, University of Ghana Medical School, College of Health Sciences, Korle-Bu, Accra, Ghana; 3 World Health Organization, Multi-Country Studies unit, Geneva, Switzerland; 4 Department of Child Health, Komfo Anokye Teaching Hospital, Kumasi, Ghana; 5 University of Newcastle Research Centre on Gender, Health and Ageing, Newcastle, Australia; 6 National Council Research, Institute of Neuroscience, Padova, Italy

**Keywords:** Edentulism, Ghana, Subjective well being, Ageing, Life satisfaction, Quality of life

## Abstract

**Background:**

Edentulism affects the quality of life and general health of an individual. But in ageing individuals, it has been observed to have greater impact, manifesting in functional, psychological and social limitations. With an increasing older adult population in Ghana, its burden is likely to increase. This study was thus carried out to explore the association between edentulism and quality of life among older Ghanaian adults.

**Methods:**

Secondary analysis of WHO’s Study on global AGEing and adult health (SAGE) Wave 1 in Ghana was conducted using self-reported edentulism as the dependent variable. Participants included a nationally representative sample of adult’s aged 50 years and older living in Ghana. Quality of life was measured using the 8 item WHOQOL measure and a single item measure which was a question “How would you rate your overall quality of life?”. To assess the association between edentulism and the independent variables, a bivariate analysis was carried out. A Poisson regression model was then performed, adjusting for age, sex, income, education and the diagnosis of a chronic disease condition. A Spearman’s correlation analysis was also carried out between the single and multi item measure of quality of life to assess how well they correlate.

**Results:**

Edentulism was observed to be associated with significantly lower levels of SWB among older adults using both the single-item and multiple-item measure (WHOQOL). It, however, showed no association with happiness. Among edentulous respondents, females and those with no formal education reported significantly lower quality of life. The WHOQOL correlated positively and strongly with the single-item measure.

**Conclusion:**

Edentulism may not be life threatening and yet it has been shown to have a negative effect on the quality of life of older adult Ghanaians. More emphasis may thus need to be placed on the oral health of the aging population in Ghana to avoid it.

## Background

Although not life threatening, the complete loss of all teeth, or edentulism, has significant impact on an individual. It has been observed to result in functional, psychological and social limitations, and affects the quality of life and general health of an individual [1].

Nutrition among these individuals may be compromised, since tooth loss affects an individual’s ability to chew effectively. It may also cause them to alter food choices and affects the digestive process [2]. Edentulism also affects the ability of the individual to speak clearly [3] and participate fully in activities due to feelings of insecurity and inferiority; this leads to considerable psycho-social problems [4]. With edentulism, facial aesthetics is also compromised; Apart from the obvious lack of teeth on opening the mouth, there is also facial sagging as a result of loss of the facial support provided by the presence of teeth, giving the individual an aged look. All of which can affect the way an individual feels about his/her life and may also act together to compromise his/her quality of life.

Tooth loss has also been associated with many chronic disease conditions e.g. diabetes, stroke, osteoarthritis, and a functional decline, all of which can be handicapping. The World Health Organization (WHO) thus considers edentulism as a poor public health outcome, which substantially affects the oral and general health status of an individual, as well as quality of life. Yet, it is often overlooked [5].

The world’s population is ageing, and the speed of ageing in middle- and low-income countries is expected to outpace that of the high-income countries [6]. The proportion of the older adult population in Ghana (a low-middle income country in West Africa) is projected to increase from 5.3% of the total population in 2014 to 8.9% by 2050 [7]. This has been attributed to better health outcomes and improved technology. With this increasing ageing population, it is important that Ghana anticipates their requirements and plans appropriate policies to address their needs, including factors that support their quality of life.

Generally the later years of life are accompanied by many physical, emotional and environmental changes that affect the quality of life of an individual. In particular, the older population in Ghana faces considerable inequality. The majority have a diminished ability to sustain themselves due to economic constraints, a generally low level of educational achievement, difficulties in access to healthcare and a loss or inversion of social roles [8]. Unfortunately this is usually the period in life when they are at a higher risk of edentulism and chronic diseases with their attendant costs, which makes them more vulnerable to factors that compromise their quality of life. To compound the problem, edentulism has also been associated with an increased risk of some of these chronic systemic diseases [9].

The way people feel about their life (subjective well-being) as they grow older is affected by social and health factors, including oral health. These to a large extent determine whether they become assets or liabilities to their families, communities and the nation. It may also affect their compliance with medical advice given by their healthcare providers since the stressful situations affecting their quality of life may make healthful actions more difficult to achieve [10].

Subjective well-being (SWB) defined as “a person’s cognitive and affective evaluations of his or her life”, is one measure of the quality of life of an individual and of societies [11]. Diener in 2000 [12] described it as a subjective definition of quality of life. He noted that it was democratic in that it grants to each individual the right to decide whether his or her life is worthwhile. The cognitive element refers to what one thinks about his or her life satisfaction in global terms (life as a whole) and in domain terms (in specific areas of life such as work, relationships, etc.) The affective element refers to emotions, moods and feelings.

Edentulism has been shown to affect an individuals’ quality of life, which then has an impact on their psychological well-being and therefore on the way they feel about their life. This effect of tooth loss on quality of life may occur as a direct result of altered function resulting from the tooth loss, or as a result of changes in perceptions and values that occur with increasing age [13]. Edentulism has also been associated with lower levels of satisfaction with life, a lower morale and self-esteem, impaired communication, and lower SWB [14-17].

In Ghana, ageing has been associated with a lower quality of life [18]. There is, however, a paucity of data on the oral health of older adults in Ghana, especially studies that assess the effect of edentulism on the quality of life of individuals and population groups. This paper seeks to explore the associations between edentulism and subjective well being among older adult Ghanaians based on nation-wide survey data. It also seeks to explore the correlation between a single-item measure and a multi-item measure in assessing SWB.

## Methods

This study was based on data from a nationally representative population of adults aged 50 years and older in Wave 1 of the World Health Organization’s (WHO) multi-country Study on Global AGEing and adult health (SAGE) Ghana [19]. SAGE Wave 1 was undertaken in Ghana in a partnership between the University of Ghana’s Department of Community Health, the Ministry of Health and WHO, as part of a multi-country longitudinal study to complement existing data on ageing to inform policy and programmes.

Face-to-face interviews were used to collect data on socio demographic characteristics, health conditions, health care utilization, satisfaction with different aspects of one’s life, tooth loss and any problems with the respondent’s mouth or teeth. Fieldwork and data entry were undertaken between May 2007 and June 2008. The World Health Organization’s Ethical Review Board approved the SAGE study, and the University of Ghana Medical School ethical and protocol review committee provided local ethical approval. Written informed consent was obtained from all study participants.

Detailed methods used for the survey including sampling, interviews, procedures for human subject protection, and consent, have been previously described [19]. All individuals who responded positively or negatively to having lost all teeth were included in this analysis.

### Measures

The dependent variable for our analysis was self-reported edentulism i.e. individuals who reported having lost all their natural teeth. Dentate individuals were those who reported to have some or all of their natural teeth present.

Independent variables for this analysis included age of the respondent, (measured as a continuous variable and categorized into four different age groups starting from age 50), sex, marital status (measured as never married, currently married, cohabiting, separated or divorced and widowed; re-categorized into living with partner and not living with a partner). Other variables included educational background, place of residence, measures of wealth (used to generate income quintiles), and religion (categorized into having a religious affiliation or not). Having ever been diagnosed with a chronic disease condition was also assessed.

The main independent variable for our analysis was SWB, which may be measured using “single-item measures” or “multi-item scales”. Generally, single-item measures are thought to be psychometrically inferior to the multi-item scales because of lower validity and reliability [20]. However, single-item measures are especially likely to be used in social surveys because they are short. Also in cross-national comparisons, single item measure (for example happiness and life satisfaction) translate well across cultures, while some of the items in multi-item scales do not.

The multi-item measure consisted of the 8-item World Health Organization Quality of Life (WHOQOL) instrument. This is a set of international, cross-culturally comparable tool used to assess quality of life that provides a measure of the evaluative component of well-being [21]. It used two questions in each of four broad domains: physical, psychological, social, and environmental [22]. Results from the eight items were summed to give an overall WHOQOL score, which was then transformed to a 0–100 scale, with lower scores indicating a better quality of life.

For the analysis of the individual items on the scale, each question had five responses: very satisfied, satisfied, neither satisfied nor dissatisfied, dissatisfied and very dissatisfied. These were re-categorized into satisfied (consisting of those who answered very satisfied and satisfied), and dissatisfied (consisting of those who answered neither satisfied nor dissatisfied, as well as those who were dissatisfied and very dissatisfied).

A single-item measure; a question “How would you rate your overall quality of life?” was also used. Responses included very good, good, moderate, bad, very bad and don’t know. The first two responses were re-categorized into good and the rest as bad. Happiness was assessed through the question “Taking all things together, how would you say you are these days? Are you very happy, happy, neither happy nor unhappy, unhappy, very unhappy or don’t know”. The first two responses were re-categorized into happy and the rest as not happy. The presence of a chronic disease condition was based on self-report by respondents through the question, “Has a health care professional ever told you, you have . . .?” The chronic disease conditions assessed included diabetes, hypertension, stroke, angina, arthritis, chronic lung disease, asthma and depression.

Wealth or income quintiles were derived from the household ownership of durable goods, dwelling characteristics (type of floors, walls, and cooking stove), and access to services (improved water, sanitation, and cooking fuel) for a total of 21 assets. A two-step random effect probit model was used to generate the quintiles. An asset ladder was first generated based on the endorsement rate of the different assets. This ladder was then used to arrange household on the same scale, based on their asset ownership. The result was a continuous income score, from which quintiles were created [7].

### Statistical analysis

Secondary data analysis was carried out using IBM SPSS version 21 statistical software. Statistical significance was set at p < 0.05. Data were summarized into absolute and relative frequencies and distribution of edentulism in the population and represented by tables. Relationships between subjective well-being and edentulism were determined using Pearson’s correlation coefficient (for association between edentulism and the single-item measure and all the eight individual items in the WHOQOL). An ANOVA test was used to assess difference in mean WHOQOL scores among edentulous and dentate respondents. A multivariate logistic regression was then carried out between edentulism and independent factors identified as significantly associated with edentulism in this study and from previous literature. Odds ratios and adjusted odds ratios and their 95% confidence intervals (CI) from simple and multiple logistic regression models were then used as an assessment of the strength of these factors. Finally, a correlation analysis was carried out between the single-item measure and the composite of the multi-item measures to test for correlation between the two.

Data on stratum sizes and household sizes for selected enumeration areas were obtained and used to calculate weights for individual respondents. Individual weights were generated using selection probabilities at each stage of selection and were poststratified by region, locality, sex, and age groups according to the 2009 projected population estimates provided by the Ghana Statistical Service.

## Results

A total of 4,724 respondents aged 50 years and older were sampled and included in this analysis. The mean age was 64.2 ± 10.73 years, with a range of 50 to 120 years. The sex composition of the sample was quite balanced, 2379 (50.4%) men and 2345 (49.6%) women.

Of the 4724 individuals interviewed, 4288 individuals responded positively or negatively to the outcome of interest (“Have you lost all of your natural teeth?”). Of these, 120 had lost all of their natural teeth, resulting in a 2.8% prevalence of edentulism.

The socio demographic characteristics of these respondents are shown in Table 1.

**Table 1:** **Background characteristics of the respondents by dental status**

Characteristic		Edentulous	Dentate
		N = 120	N = 4168
		n (%)	n (%)
**Sex**			
	Female	68 (56.7)	1953 (46.9)
	Male	52(43.3)	2215 (53.1)
**Age**			
	50-59	26 (21.7)	1656 (39.7)
	60-69	28 (23.3)	1175 (28.2)
	70-79	28 (23.3)	950 (22.8)
	80+	38 (31.7)	387 (9.3)
**Marital status**			
	Living with partner	48 (40.3)	2373 (57.2)
	Living without partner	71 (59.7)	1773 (42.8)
**Education**			
	No formal education	77 (64.7)	2196 (53.0)
	Some formal education	42 (35.2)	1948 (47.0)
**Location of residence**			
	Urban	63 (52.2)	1690 (40.5)
	Rural	57 (47.5)	2478 (59.5)
**Income quintile**			
	Q1 (poorest)	25 (20.8)	829 (19.9)
	Q2	23 (19.2)	824 (19.8)
	Q3	24 (20.0)	831 (20.0)
	Q4	22 (18.3)	845 (20.3)
	Q5 (richest)	26 (21.7)	834 (20.0)
**Problems with mouth/teeth**			
	Yes	36 (30.0)	396 (9.5)
	No	84 (70.0)	3771 (90.5)
**Oral healthcare utilization**			
	Dental attendance past 2 weeks	8 (22.2)	21 (5.3)
	Dental attendance past 12 months	9 (25.0)	61 (15.4)
**Religious affiliation**			
	Yes	115 (95.8)	3937 (94.9)
	No	5 (4.2)	211 (5.1)
**Diagnosis of a chronic condition**			
	Yes	62 (51.7)	1388 (33.3)
	No	58 (48.3)	2780 (66.7)

Edentulism was observed to be positively associated with age, the female gender, those living without a partner, those with no formal education, those resident in an urban area and those with a known diagnosis of a chronic disease (Table 2).

**Table 2:** **Socio-demographic characteristics of respondents and their association with edentulism in a bivariate logistic regression analyses**

Characteristic	Number n (%)	Crude Odds Ratio (95% CI)	P – value
**Sex**			
Female	68 (56.7)		
Male	52 (43.3)	0.674 (0.468-0.972)	0.034
**Age group**			
50-59	26 (21.7)	0.160 (0.096-0.261)	<0.001
60-69	28 (23.3)	0.243 (0.147-0.401)	<0.001
70-79	28 (23.3)	0.300 (0.182-0.496)	<0.001
80+	38 (31.7)		
**Marital status**			
Living with partner	48 (40.3)		
Living without partner	71 (59.7)	1.980 (1.366-2.870)	<0.001
**Educational status**			
No formal education	77 (64.7)	1.626 (1.111-2.380)	0.012
Formal education	42 (35.3)		
**Location of residence**			
Urban	63 (52.2)	1.621 (1.127-2.331)	0.009
Rural	57 (47.5)		
**Income quintile**			
Q1 (Poorest)	25 (20.8)	0.967 (0.554-1.689)	0.907
Q2	23 (19.2)	0.895 (0.507-1.582)	0.704
Q3	24 (20.0)	0.926 (0.528-1.627)	0.790
Q4	22 (18.3)	0.835 (0.470-1.485)	0.540
Q5 (Richest)	26 (21.7)		
**Religious affiliation**			
Yes	115 (95.8)	1.233 (0.498 – 3.050)	0.651
No	5 (4.2)		
**Known diagnosis of chronic disease**			
Yes	62 (51.7)	2.141 (1.488 -3.080)	<0.001
No	58 (48.3)		
**Problems with mouth/teeth**			
Yes	36 (30.0)	4.08 (2.73 – 6.11)	<0.001
No	84 (70.0)		
**Oral healthcare utilization**			
Dental attendance (past 2 weeks)	8 (22.2)	5.088 (2.068-12.520)	0.000
Dental attendance (past 12 months)	9 (25.0)	1.825 (0.818-4.071)	0.137

Edentulous individuals reported lower levels of subjective well-being than dentate individuals (Table 3). Being edentulous was also significantly associated with a report of dissatisfaction with seven out of the eight individual measures for subjective well-being that formed the WHOQOL in a bivariate analysis. The only item that didn’t differ between the two groups was the question “Do you have enough money to meet your needs?”

**Table 3:** **Subjective well-being and quality of life by dental status in a bivariate and multivariate analysis**

Satisfaction	Edentulous	Dentate	OR (95% CI)	AOR (95% CI)
	n (%)	n (%)		
**Do you have enough energy for everyday life**				
Yes	23 (19.2)	1295 (31.1)	1.9 (1.205 - 3.020)***	1.3 (0.760 -2.021)
No	97 (80.8)	2863 (68.9)		
**Do you have enough money to meet your needs**				
Yes	6 (5.1)	262 (6.3)	1.3 (0.550 - 2.897)	0.83 (0.356 – 1.935)
No	112 (94.9)	3875 (93.7)		
**Your health**				
Satisfied	36 (30.0)	2385 (57.3)	3.1 (2.110 - 4.654)***	0.42 (0.278 -0.637)***
Not satisfied	84 (70.0)	1776 (42.7)		
**Yourself**				
Satisfied	61 (50.8)	2826 (68.0)	2.1 (1.426 – 2.952)***	0.64 (0.438 – 0.946)*
Not satisfied	59 (49.2)	1332 (32.0)		
**Your ability to perform daily activities**				
Satisfied	43 (35.8)	2421 (58.2)	2.5 (1.711 -3.645)***	0.56 (0.374 – 0.838)**
Not satisfied	77 (64.2)	1736 (41.8)		
**Your personal relationships**				
Satisfied	76 (63.3)	3135 (75.4)	1.8 (1.218 – 2.594)**	0.76 (0.511 – 1.137)
Not satisfied	44 (36.7)	1021 (24.6)		
**Your living conditions**				
Satisfied	61 (50.8)	2488 (59.9)	1.5 (1.006 – 2.080)*	0.72 (0.493 – 1.050)
Not satisfied	59 (49.2)	1664 (40.1)		
**Taking all things together, how satisfied are you with your life**				
Satisfied	44 (36.7)	2361 (56.8)	2.3 (1.561 – 3.314)***	0.53 (0.357 – 0.789)**
Not satisfied	76 (63.3)	1793 (43.2)		
**How would you rate your overall quality of life (Single item measure)**				
Satisfied	25 (20.8)	1244 (30.0)	1.6 (1.043 – 2.542)*	0.76 (0.478 – 1.212)
Not satisfied	95 (79.2)	2904 (70.0)		
**How happy are you these days?**				
Happy	72 (61.0)	2491 (62.1)	1.0 (0.720 – 1.525)	1.16 (0.788 – 1.715)
Not happy	46 (39.0)	1519 (37.9)		

*p < 0.050 **p < 0.010 ***p < 0.001.

After adjusting for age, sex, income, education and the diagnosis of a chronic disease condition, four out of the eight individual measures remained significantly associated with edentulism.

The composite WHOQOL score showed differences between the edentulous and dentate respondents; (Table 4). The edentulous subjects recording a higher mean WHOQOL score than dentate subjects.

**Table 4:** **Mean WHOQOL scores for dentate and edentulous respondents**

Dental status	Mean WHOQOL	SD	p-value
Dentate	54.09	±13.661	0.00
Edentulous	61.60	±15.543	

With regard to the single-item measure, edentulous respondents reported not being satisfied with their overall quality of life compared with the dentate respondents. After adjusting for age, sex, income, education and the diagnosis of a chronic disease condition, however, this difference ceased to be significant.

In relation to happiness there was no difference between edentulous and dentate respondents.

Being edentulous was significantly associated with a higher mean WHOQOL score thus a higher level of dissatisfaction with one's life.

Among edentulous respondents, females and those with no formal education reported significantly higher WHOQOL scores (Table 5).

**Table 5:** **Mean WHOQOL scores for edentulous respondents by sociodemographic characteristics**

Characteristic	Number n (%)	Mean WHOQOL scores ± SD	P – value
**Sex**			
Female	68 (56.7)	64.67 ± 15.15	0.013*
Male	52 (43.3)	57.59 ± 15.29	
**Marital status**			
Living with partner	48 (40.3)	58.23 ± 15.54	0.065
Living without partner	71 (59.7)	63.56 ± 15.13	
**Educational status**			
No formal education	77 (64.7)	65.55 ± 15.63	< 0.001*
Formal education	42 (35.3)	53.81 ± 11.97	
**Location of residence**			
Urban	63 (52.2)	60.83 ± 16.39	0.570
Rural	57 (47.5)	62.46 ± 14.65	
**Religious affiliation**			
Yes	115 (95.8)	61.35 ± 15.25	0.388
No	5 (4.2)	67.50 ± 22.64	
**Known diagnosis of chronic disease**			
Yes	62 (51.7)	62.58 ± 15.70	0.479
No	58 (48.3)	60.56 ± 15. 44	

A Spearman’s correlation analysis carried out between the single-item measure of quality of life and the composite of all the responses (WHOQOL) score demonstrated that the single item measure correlated positively and strongly well with the composite WHOQOL score. The correlation coefficient was 0.703 (p = 0.000).

## Discussion

There is a growing interest in dentistry to understand the patient’s perception of oral health and disease and its linkage to their psychology [23]. Teeth have social, psychological and cultural significance due to their importance in verbal and nonverbal communication [24]. Their loss, therefore, is often perceived as traumatic and has been associated with a loss of vitality [1]; this may affect the way an individual perceives his or her life.

From this analysis, edentulism was found to be associated with significantly lower levels of SWB and satisfaction with life from this analysis. This is consistent with other studies where edentulism was also associated with lower levels of SWB, satisfaction with life, a lower morale and self-esteem and impaired communication [14-17].

In this study, edentulism showed an association with lower levels of SWB and self reported quality of life. It also showed lower levels of satisfaction for almost all the proxy measures of quality of life. This finding is consistent with several other studies [23,25-27]. Brennan et al. [28] noted that edentulism has an affect on an individuals’ quality of life which then has an impact on their psychological well-being and therefore on their SWB. This effect of tooth loss on quality of life may occur as a direct result of altered function resulting from the tooth loss, or as a result of changes in perceptions and values that occur with increasing age [13]. The number of functional teeth has been shown to be positively associated with chewing ability [28]; and chewing ability has also been associated with oral-health-related quality of life and general health. This effect of edentulism on mastication has resulted in most edentulous individuals having to alter their diet, which results in a negative impact on the enjoyment of meals, and also on the quantities and types of nutrients consumed. Edentulous individuals tend to favor softer, more processed foods, which are typically higher in fat and cholesterol content [29] and may also be lacking in vitamins and minerals. Thus, being edentulous may predispose one to comorbidities due to difficulty in obtaining sufficient nutrition intake to maintain good general health.

Social relationships have been implicated as a causal factor for health [30] and there is an inverse association between social relationships and psychological ill health, cognitive disability, cardiovascular disease, [31] and mortality [32]. Individuals belonging to social networks are more likely to follow health-enhancing behaviors and to have higher self-esteem and, hence, have better health [33].

Secondly, social relationships tend to buffer the negative effects of stressors on health, as individuals with better social support have wider access to information, financial resources, and emotional resources that help mitigate consequences of stressful events. Such individuals are also better at altering their behaviors to cope with diseases and risk factors [34]. Studies have shown that social relationships have a protective effect on oral health. In adults, social support has been observed to be a determinant of oral health-related quality of life. Psychosocial factors, such as loneliness and social isolation, have also been associated with the onset of periodontal disease [35]. In this study, edentulous respondents were less satisfied with their personal relationships in a bivariate analysis. After adjusting for age, sex, education, diagnosis of a chronic disease and income, however, this association ceased to be significant. This may be accounted for by the fact that among older adults, poorer social support has been found to be associated with having fewer functioning teeth, worse dental behaviors, and more periodontal attachment loss [36,37]. Tsakos et al. [38] observed that having good social support was associated with better self-rated oral health, and more teeth. Furthermore, individuals tended to avoid social interactions due to embarrassment and/or functional problems with perceived inadequate dentition [39,40]. People without natural teeth also tend to avoid close relationships because they fear rejection when the other party discovers their edentulism [41].

Edentulous respondents were also less satisfied with their general health, a finding consistent with other studies [28].

Steele [13] also observed that subjective well-being has been linked to self-rated oral health among older adults.

Among edentulous respondents, factors that were observed to influence SWB included sex, age, education and income, with edentulous females and those with no formal education reporting lower levels of SWB. This finding is consistent with another study in Ghana [18] where these factors also influenced SWB among the general Ghanaian population.

Happiness, on the other hand, showed no association with edentulism. This might be because people’s moods and emotions reflect reactions to events happening to them. Life satisfaction and morale are conceptualized as relatively stable orientations toward life that, though evaluative, are not affected by transient moods. Happiness on the other hand is viewed as less stable and less cognitive than life satisfaction and positive affect is expected to be the least stable, changing rapidly and frequently in response to stimuli in the immediate environment [42].

This study also assessed the correlation between the single-item and the multi-item measures in evaluating SWB in the Ghanaian setting. Our results showed that the two correlated very well.

Generally, single-item measures are usually psychometrically inferior to multi-item and multidimensional scales because of lower validity and reliability. That does not seem to be true, however, for single-item measures of subjective well being [43]. Furthermore, psychometric evaluations of these simple scales showed that they possessed a degree of validity. Again, Andrews & Crandall [44] found that global questions about people’s overall evaluation of their lives yielded scores that converged well with one another. Abdel-Khalek [45], in examining the accuracy of measuring subjective well-being with a single-item measure, concluded that its temporal stability was 0.86 (44). Furthermore, correlation between the single-item and multi-item scales were highly significant and positive, denoting good concurrent validity.

This study had certain limitations that may require a cautious interpretation of the results. The SAGE Wave 1 relied on self-reported individual submissions and did not objectively confirm the diagnosis of being edentulous or dentate. This may have resulted in an underestimation of prevalence rates compared to measured rates. Information on denture use was also not assessed, a factor that may have had an influence of the quality of life of the subjects. The analysis, however, provided information on the prevalence of self-reported edentulism and SWB among older persons in Ghana as a baseline for further investigations.

## Conclusion

Edentulism was observed to be associated with significantly lower levels of SWB among older adults using both the single-item and multiple-item measures (WHOQOL). The WHOQOL correlated positively and strongly with the single-item measure. Unfortunately, edentulism has been largely overlooked and hasn’t been seen as a huge priority in managing ageing health issues in Ghana. It however, takes a toll on the quality of life of the individual and oral care for older adults should aim to preserve the natural teeth where possible, since their preservation can help to enhance a positive body image and self- worth, and positively influence quality of life. The government may need to consider incorporating oral health policies into its National Ageing Policy document to guide the management of the aged in Ghana.

## Abbreviations

AOR: Adjusted odds ratio
OR: Odds ratio
SAGE: Study on global ageing and adult health
SWB: Subjective well-being
WHO: World Health Organization
WHOQOL: World Health Organization Quality of Life
